# Supplementary material for: Microcystin Concentrations, Partitioning, and Structural Composition during Active Growth and Decline: A Laboratory Study
Source: Toxins (Basel). 2023 Dec 6;15(12):684. doi: 10.3390/toxins15120684 (PMC10746996; doi:10.3390/toxins15120684)
Supplement: Supplementary file 1 [file toxins-15-00684-s001.zip › toxins-2719585-supplementary.pdf]

Supplementary Materials: Microcystin Concentrations, Partitioning, and Structural Composition during Active Growth and Decline: A Laboratory Study

Table S1. Sampling sites along the Chowan River, NC.

Site	Full Name	Date	Latitude	Longitude
**AH**	Arrowhead	07/16/2019	36.23	-76.71
**CR**	Colerain	07/16/2019	36.20	-76.75
**IR**	Indian River	07/31/2019	36.23	-76.70
**LL**	Leary's Landing	07/31/2019	36.14	-76.75
**MC**	Modoc Canal	08/13/2019	36.22	-76.71
**CP**	Charlton Pier	09/11/2019	36.14	-76.75

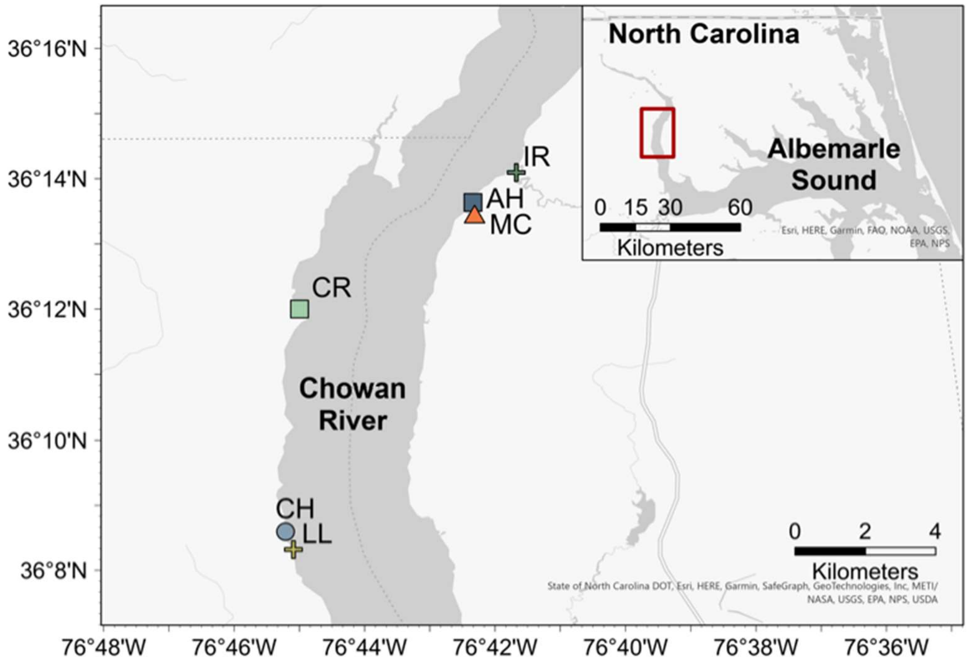

Figure S1. Map of sampling sites in Chowan River, NC (AH = Arrowhead; CR = Colerain; IC = Indian Creek; LL = Leary's Landing; MC = Modoc Canal; CP = Charlton Pier).
